# An arctic breeding songbird overheats during intense activity even at low air temperatures

**DOI:** 10.1038/s41598-024-65208-9

**Published:** 2024-07-02

**Authors:** Ryan S. O’Connor, Oliver P. Love, Lyette Régimbald, Audrey Le Pogam, Alexander R. Gerson, Kyle H. Elliott, Anna L. Hargreaves, François Vézina

**Affiliations:** 1 Département de Biologie, Chimie et Géographie, Université du Québec à Rimouski, 300 Allée des Ursulines, Rimouski, QC G5L 3A1 Canada; 2 Groupe de Recherche sur les Environnements Nordiques BORÉAS, Rimouski, Canada; 3 Centre d’études Nordiques, Rimouski, Canada; 4 Centre de la Science de la Biodiversité du Québec, Rimouski, Canada; 5 Department of Integrative Biology, University of Windsor, Windsor, ON N9B 3P4 Canada; 6 Department of Biology, University of Massachusetts Amherst, Amherst, MA 01003 USA; 7 Department of Natural Resource Sciences, McGill University, Sainte Anne de Bellevue, QC H9X 3V90 Canada; 8 Department of Biological Sciences, McGill University, Montréal, QC H3A 1B1 Canada

**Keywords:** Arctic breeding species, Climate change, Evaporative cooling, Flight, Heat tolerance, Hyperthermia, Thermoregulation, Ecophysiology, Animal physiology, Physiology

## Abstract

Birds maintain some of the highest body temperatures among endothermic animals. Often deemed a selective advantage for heat tolerance, high body temperatures also limits birds’ thermal safety margin before reaching lethal levels. Recent modelling suggests that sustained effort in Arctic birds might be restricted at mild air temperatures, which may require reductions in activity to avoid overheating, with expected negative impacts on reproductive performance. We measured within-individual changes in body temperature in calm birds and then in response to an experimental increase in activity in an outdoor captive population of Arctic, cold-specialised snow buntings (*Plectrophenax nivalis*), exposed to naturally varying air temperatures (− 15 to 36 °C). Calm buntings exhibited a modal body temperature range from 39.9 to 42.6 °C. However, we detected a significant increase in body temperature within minutes of shifting calm birds to active flight, with strong evidence for a positive effect of air temperature on body temperature (slope = 0.04 °C/ °C). Importantly, by an ambient temperature of 9 °C, flying buntings were already generating body temperatures ≥ 45 °C, approaching the upper thermal limits of organismal performance (45–47 °C). With known limited evaporative heat dissipation capacities in these birds, our results support the recent prediction that free-living buntings operating at maximal sustainable rates will increasingly need to rely on behavioural thermoregulatory strategies to regulate body temperature, to the detriment of nestling growth and survival.

## Introduction

Birds maintain high and relatively constant body temperatures (e.g., 38–41 °C) across a wide range of environmental temperatures^1–3^. When heat stressed, birds often allow body temperature to increase above normothermic levels (i.e., facultative hyperthermia), thereby aiding in water conservation by simultaneously augmenting the thermal gradient between body and air temperature and delaying the onset of evaporative water loss that would result from active cooling^4–6^. However, as birds already operate at high body temperatures, their capacity for increasing body temperature above normothermic levels is limited before reaching potentially lethal body temperatures of 45–47 °C^7,8^. Consequently, birds working at peak metabolic levels (e.g., nestling-provisioning adults) under high heat loads regularly increase their evaporative heat dissipation behaviours and/or reduce activity to limit endogenous heat production and avoid lethal body temperatures^9,10^. Individuals are therefore presented with a trade-off during periods of elevated ambient temperatures (T_a_) wherein they must increase thermoregulatory behaviours while decreasing other essential activities, culminating in possible costs to body condition, reproduction, and survival^11,12^. For example, van de Ven et al.^13^ showed that the probability of successful fledging among southern yellow-billed hornbills (*Tockus leucomelas*) fell below 50% when maximum air temperatures rose above 35 °C. Moreover, nestling hornbills exposed to the hottest weather fledged the nest 50% lighter than those raised under the coolest nesting conditions, likely due in part to decreased provisioning rates of the male hornbill at high maximum air temperatures. Thus, the complex interaction among environmental temperature, activity, and body temperature regulation can have pronounced impacts on the quality of parental care among avian species (see review by^12^).

The Arctic is warming rapidly, at rates 3–4 times the global average^14^. These thermal changes have already affected critical ecological processes^15–17^, due to phenological mismatches^18,19^, increased parasitism^20,21^ and invasive species^22,23^. In contrast, the direct impacts of rapidly increasing temperatures on the behaviour and physiology of Arctic species is less well understood, despite Arctic species being potentially significantly at risk given their evolved traits for conserving heat and coping with extreme cold^24^. As such, Arctic cold-speclialists may now be facing signifcant performance constraints in a rapidly warming Arctic due to co-adaptations to perform optimally under cooler conditions, but at the expense of reduced performance at warmer temperatures^25,26^. Indeed, recent research on Arctic birds suggests comparatively high heat sensitivities, with individuals exhibiting signs of active heat dissipation at relatively moderate air temperatures and/or low body temperatures^27,28^. Moreover, Arctic birds’ capacity for physiological thermoregulation through increased evaporative water loss appears significantly constrained^27,28^. Indeed, recent thermal modelling predicts that under increasing air temperatures, cold-adapted Arctic birds will have to allocate more time toward body temperature regulation through behavioral adjustments to avoid lethal hyperthermia, at the expense of essential activities (e.g., provisioning)^29^.

Snow buntings (*Plectrophenax nivalis*; hereafter ‘buntings’) are Arctic-breeding songbirds that spend most of their life in sub-zero air temperatures^30,31^. Even on their breeding grounds, buntings can experience blizzard conditions and air temperatures of − 25 °C^30^. Consequently, buntings are cold-specialists that can tolerate air temperatures equivalent to − 94 °C under laboratory conditions^32^. In contrast, buntings are constrained in their capacity to dissipate heat through evaporative pathways and become heat stressed at relatively moderate air temperatures^28^. As a result, at warmer environmental temperatures buntings are expected to rely on behavioural thermoregulatory strategies to maintain non-lethal body temperatures^29^.

To investigate body temperature variation and heat dissipation behaviours in snow buntings in relation to thermal conditions, we worked with an outdoor captive population and took advantage of the natural seasonal variation in air temperatures from − 15 (winter) to 36 °C (summer). We measured body temperature patterns in calm birds to first establish baseline inter-individual variation in body temperature, from which we experimentally determined individual spare capacity for increases in body temperature up to lethal levels (i.e., 45–47 °C) when actively flying. We quantified these activity-based increases in body temperature relative to air temperature and also quantified resulting heat dissipation behaviours (e.g., panting or wing spreading). We expected maximum body temperatures in actively flying buntings to increase with air temperature, with buntings showing signs of heat stress via panting at comparatively low air temperatures. We were particularly interested in determining at which air temperatures actively flying buntings would begin to exhibit body temperatures approaching or exceeding lethal levels, as breeding buntings may increasingly have to rely on behavioural trade-offs under a warming Arctic to avoid high body temperatures^29^.

## Materials and methods

### Ethical statement

All bird handling was approved by the Animal Care Committee of the Université du Québec à Rimouski (CPA-81-20-223 and CPA-71-17-194) and was conducted under an Environment and Climate Change Canada scientific permit (SC-48).

### Body and air temperature measurements in calm birds

Buntings were captured on farmlands close to Rimouski, Québec, Canada, and maintained in an outdoor aviary at the Université du Québec à Rimouski as described by Le Pogam et al.^32^. Birds were maintained in an outdoor aviary (5.8 mW × 5.3 mD × 2.6 m to 3.6 mH, angled ceiling), under naturally-changing photoperiod conditions, and were exposed to the elements while being sheltered from direct precipitation. Birds were fed ad libitum with a seed-mix (crushed corn, wheat, sorghum, white millet, red millet, black oil sunflower; Armstrong, Hagersville, ON, Canada) and Mazuri Small Bird Maintenance Mini Diet (#562A; Mazuri, Richmond, IN, USA) using several poultry feeders to guarantee access to food for all individuals. We also offered water ad libitum using automatic heated bowls to prevent freezing in winter (1L, Ukal, St-Hyacinthe, QC, Canada). The water was supplemented with electrolytes four days per week (0.2g/L; Electrolytes Plus, Vetoquinol N.-A. Inc. Lavaltrie, QC, Canada) and vitamins three days per week (0.6g/L; Poly-tonine A Complex, Vetoquinol N.-A. Inc. Lavaltrie, QC, Canada).

We implanted buntings with a temperature-sensitive, passively integrated transponder (PIT) tag (Biotherm 13, Biomark, Boise USA; published read range 33–43 °C; ± 0.5 °C; actual read limit with same confirmed accuracy to 50 °C). These non-archival tags return a tag number and temperature reading each time the tag is activated by the antenna of a data logger (HPR Plus, Biomark, Boise USA). We implanted 24 birds with PIT tags subcutaneously either under the left wing on their flank (n = 6; Nord et al.^33^), or on their neck between the scapulae (n = 18; Oswald et al.^34^). We also implanted 19 birds with PIT tags intraperitoneally (Whitfield et al.^35^). Using these 43 captive buntings, we measured body temperature patterns in calm (i.e., normal activity) birds over 3 periods: 4 June to 12 December 2019, 28 May to 15 November 2020, and 4 March to 16 September 2021. There was no evidence for a difference in temperature between subcutaneous flank and neck implant locations (*P* = 0.63; mean ± SD flank T_b_ = 41.0 ± 1.1 °C; neck T_b_ = 41.2 ± 0.7 °C). Consequently, we combined these two locations into one subcutaneous group. Body temperature was recorded every time birds approached their feeder by placing a racket antenna underneath the food source which was attached to a data logger that scanned for body temperature at 10 s intervals. Air temperature was recorded at 30 min intervals using iButton data loggers (DS1922L, Maxim Integrated, San Jose USA) suspended 2 m off the ground in the shade.

### Body and air temperature measurements in active birds

To investigate how air temperature influences maximum body temperatures in active snow buntings, we conducted 36 heat stress experiments on 32 actively flying snow buntings from May through December of 2019 and 2020. On the morning of experiments, we measured the body mass of each bunting using a digital scale. We timed experiments to coincide with the hottest part of the day, with an average start time of 15:04 h (range = 14:02–16:03 h). When we entered the aviary, buntings began to fly naturally to avoid capture and therefore experiments started upon entering the aviary (i.e., minute = 0). We captured buntings in flight at various times following minute 0 (range = 32 s to 33 min) with a butterfly net. Upon capture, we immediately measured the body temperature with a racket antenna and data logger (HPR Plus, Biomark, Boise USA) and subsequently released the bird into an adjoining aviary. The average duration of experiments was 23 ± 4 min (range = 19–33 min). If buntings had a body temperature ≥ 45 °C we immediately submerged their bodies from the neck down in cold water prior to releasing them to aid in evaporative cooling. Heat stress experiments ended after the last bunting was released and we left the aviary. Air temperature was measured using the same iButton loggers as for calm birds.

### Data analyses

We performed analyses in R version 4.1.0 (R Core Team 2022). Reported values are mean ± standard deviation (SD), where means represent the combined average across individual bird averages. For example, mean subcutaneous body temperature was calculated by taking a mean body temperature for each bird with a subcutaneous implant and then calculating the combined average across birds. We only included body and air temperature values measured between sunrise and sunset for each day (i.e., diurnal body temperatures). We calculated sunrise and sunset times within the *suncalc* package^36^. Additionally, to avoid possible confounds of additional stress we removed body temperatures recorded during periods when food was replaced and the 10 min period after a person left the aviary. To facilitate comparisons between body and air temperature in calm snow buntings, we rounded times to the nearest half-hour. For example, if body or air temperature was measured at 10:44 it was rounded down to 10:30, whereas a recording at 10:45 was rounded up to 11:00. We then averaged body temperature to the nearest 1 °C air temperature. For example, mean body temperature at an air temperature of 20 °C included all body temperature values measured between air temperatures of 19.5 and 20.4 °C. We calculated variation in bunting body temperature around their modal body temperature using Boyles et al.^37^, heterothermy index (HI):

$$HI=\sqrt{\frac{\sum {(T}_{b-mod}-{T}_{b-i}{)}^{2}}{n-1}}$$

where T_b-mod_ is the modal body temperature in calm birds during daylight hours, T_b-i_ is the body temperature measured at time i, and n is the number of times body temperature was sampled. Given that modal body temperature represents the most frequently occurring body temperature, we follow Boyles et al.^37^ and assume this value to represent the optimal body temperature for performance in buntings.

To compare mean body temperature metrics (i.e., mean body temperature, mean modal body temperature, mean maximum body temperature, and mean HI) among subcutaneous and intraperitoneally implanted buntings, we performed simple linear regression within the *stats* package (R Core Team 2022). When reporting *P*-values, we follow the guidelines of Muff et al.^38^ and present our results in terms of strength of evidence rather than terms of significance. Additionally, we report parameter estimates from all models along with *P*-values.

To determine variation in body temperature patterns among active buntings, we used linear mixed-effect models within the *lme4* package^39^. Bird identity was included as a random factor due to repeated sampling from the same individuals. We first built a global model with the following fixed input variables: air temperature (T_a_), body mass (M_b_), intraperitoneal *vs.* subcutaneous tag location (IP/SQ) and activity duration (i.e., the time T_b_ was measured in minutes relative to entering the aviary). We also included the two-way interactions between T_a_:M_b_, T_a_:IP/SQ, T_a_:Activity duration, and M_b_:Activity duration, as well as the 3-way interaction between T_a_:M_b_:Activity duration. Prior to fitting the model, we mean-centered and standardized the continuous input variables by dividing by 2 standard deviations^40^ using the standardize function within the *arm* package^41^. We performed model selection on the global model using the dredge function within the *MuMIn* package^42^ and selected the 95% confidence set of the best-ranked regression models (i.e., all the top models whose cumulative Akaike weight is 0.95, where Akaike weight represents the probability that a given model is the best approximating model; Symonds and Moussalli^43^).We then averaged the model parameter estimates from the 95% confidence set. We derived slopes from the model averaged parameter estimates using the ggpredict function within the *ggeffects* package^44^ to calculate estimated marginal means for the response variable. During the 2020 sampling period, we also aimed to quantify the percentage of birds panting relative to air temperature by recording the presence or absence of panting in buntings upon release into the adjoining aviary. Lastly, we calculated the mean hyperthermic scope (T_b_ − T_b-mod_) of indiviual active buntings by subtracting an individual’s measured T_b_ at time of capture from their modal T_b_. All figures were produced using ggplot2^45^.

## Results

### Body temperature patterns in calm birds

**Figure 1:**
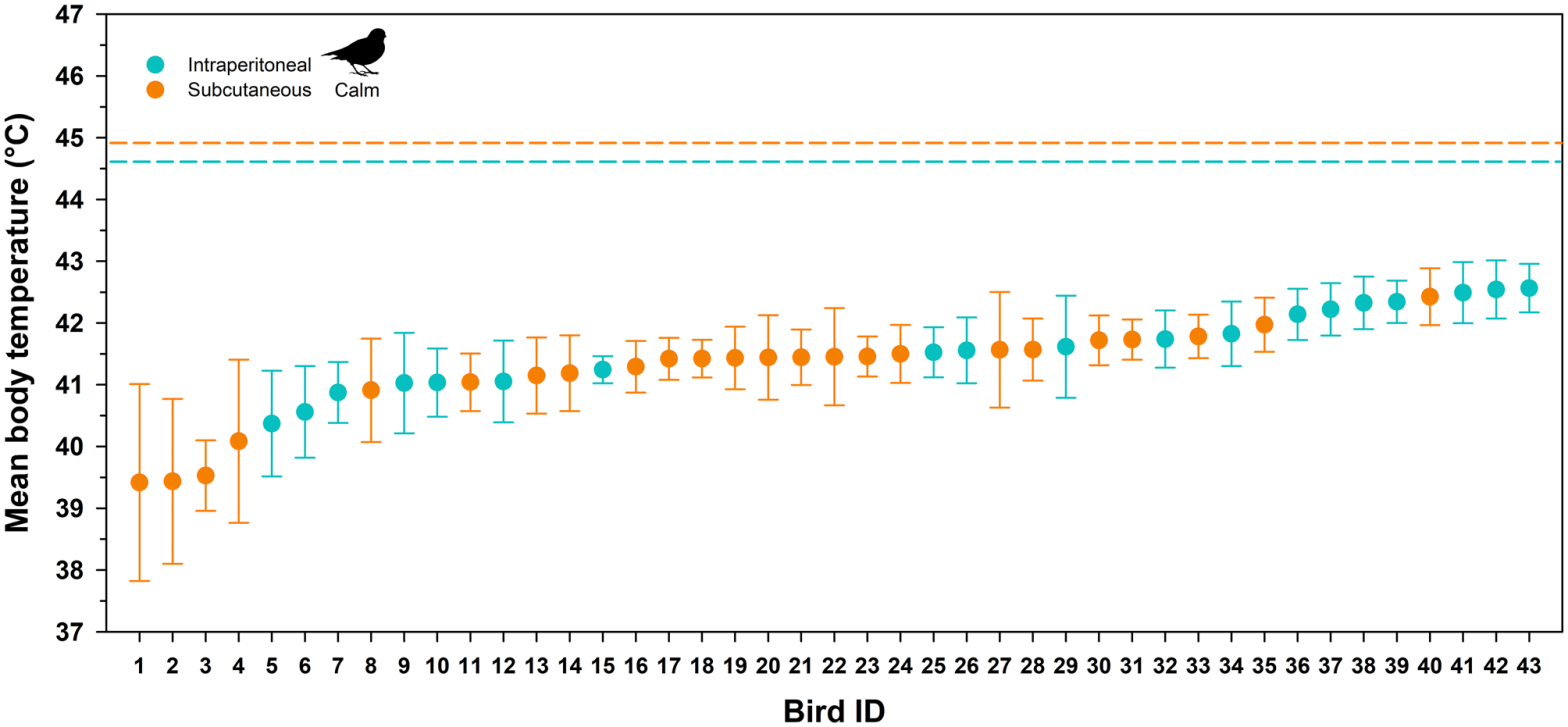
Individual mean body temperatures for calm (non-active) snow buntings (*Plectrophenax nivalis*) implanted intraperitoneally or subcutaneously with temperature sensitive transponder tags. Error bars represent standard deviation. The horizontal dashed lines are the respective mean maximum body temperatures for intraperitoneal and subcutaneous birds when actively flying. All values represent data recorded between sunrise and sunset.

**Figure 2:**
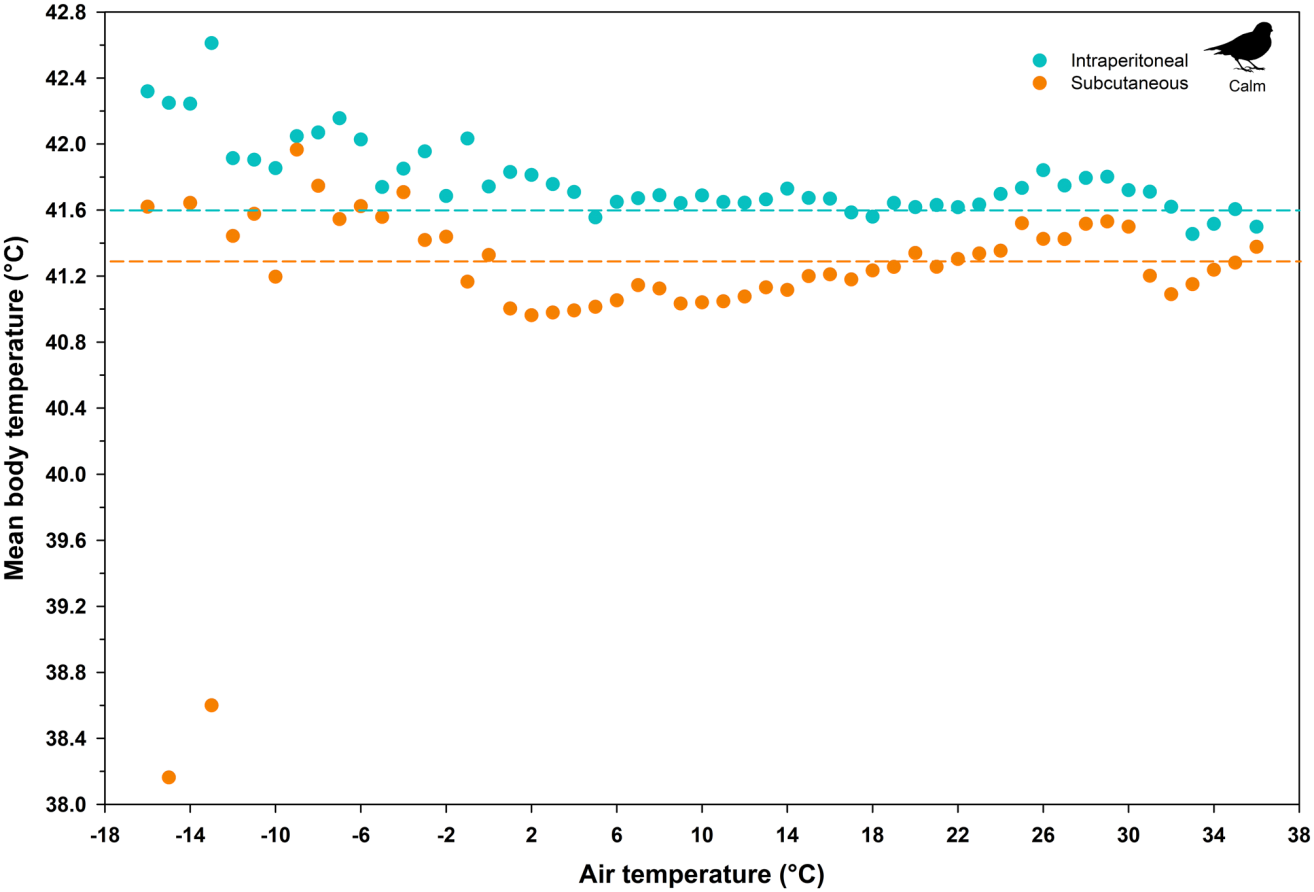
Mean body temperature as a function of air temperature measured in a captive population of calm (non-active) snow buntings (*Plectrophenax nivalis*). Horizontal lines represent the respective average modal body temperatures for intraperitoneal and subcutaneous birds. Error bars were removed for clarity. All values represent data recorded between sunrise and sunset.

There was substantial inter-individual variation in mean body temperature in calm birds, ranging from 39.4 to 42.6 °C (Fig. 1). In calm birds, body temperatures for both intraperitoneal and subcutaneous implanted individuals increased above modal body temperature at lower air temperatures (Fig. 2). On 19 occasions, 13 different calm birds (9 intraperitonealand 4 subcutaneous) had body temperatures ≥ 45°C (Table 1). We found only weak evidence for differences in mean body temperature among intraperitonal and subcutaneous tags (Table 1). However, on average intraperitoneal birds had higher maximum body temperatures (Table 1).

**Table 1:** Mean ± standard error in body temperature (T_b_), modal body temperature (T_b-mod_), maximum body temperature (T_b-max_) and heterothermy index (HI) across calm snow buntings (*Plectrophenax nivalis*) with intraperitoneal or subcutaneous implants.

Variable	Intraperitoneal	Subcutaneous	Estimate ± SE	*P-*value
Mean T_b_	41.6 ± 0.7 °C (40.4–42.6 °C)^a^	41.2 ± 0.8 °C (39.4–42.4°C)	0.452 ± 0.229	0.055
Mean T_b-Mod_	41.6 ± 0.8 °C (39.9–42.6 °C)	41.3 ± 1.1 °C (37.7–42.5 °C)	0.329 ± 0.294	0.270
Mean T_b-max_	46.0 ± 3.2 °C (41.9–51.9 °C)	44.0 ± 2.6 °C (42.1–51.6 °C)	1.957 ± 0.889	0.034
Mean HI	0.57 ± 0.26 °C (0.33–1.44 °C)	0.60 ± 0.39 °C 0.31–1.97 °C)	-0.035 ± 0.104	0.737

All values represent diurnal body temperatures recorded between sunrise and sunset. Values in parentheses represent the range of values for individual birds.

^a^Represents the range of mean body temperatures for individiual birds.

### Body temperature patterns in active birds

**Figure 3:**
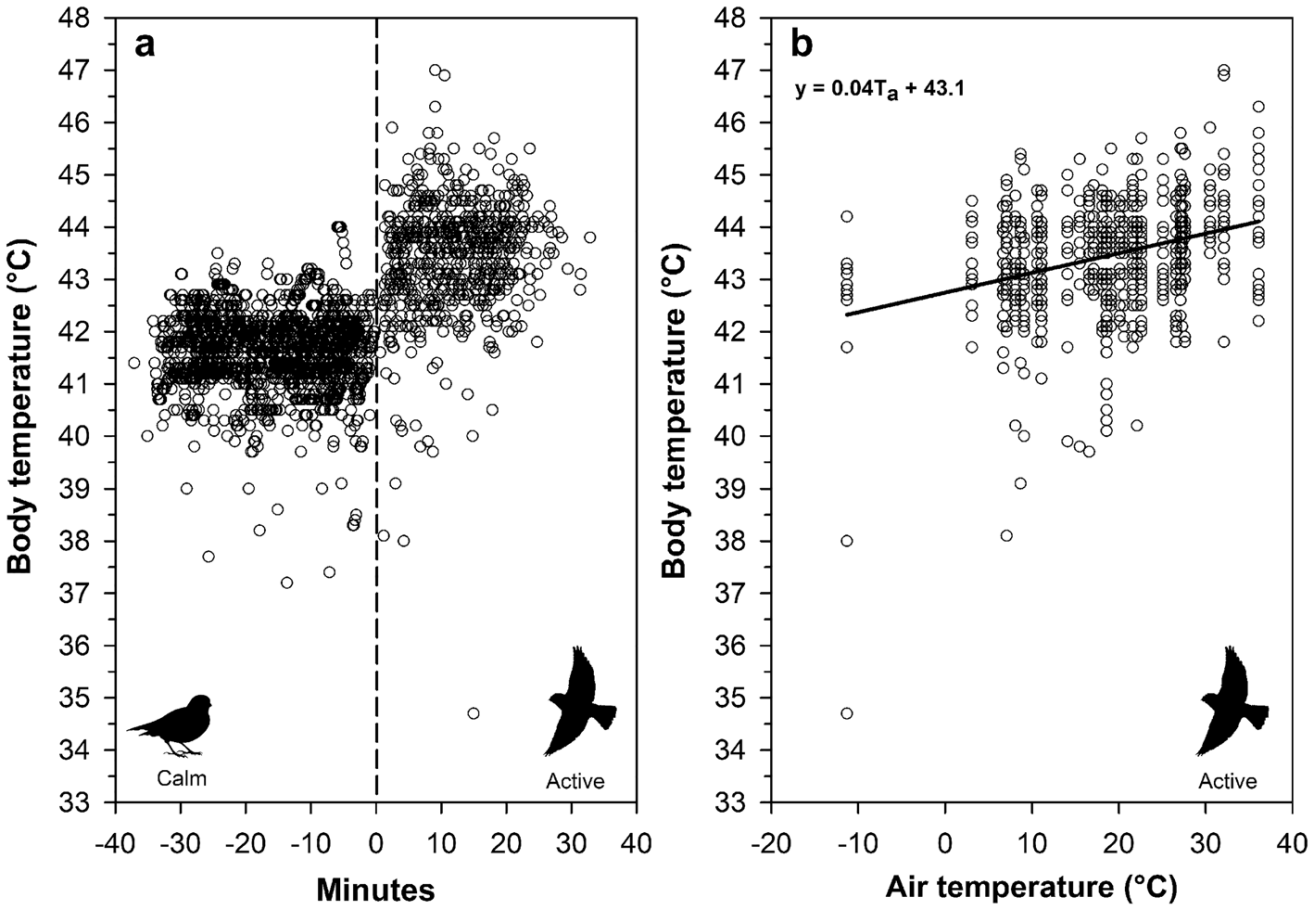
Body temperature patterns in active snow buntings (*Plectrophenax nivalis*) as a function of **a** time since entering the aviary and **b** air temperature. In panel a, negative minutes represent the time span prior to entering the aviary and positive values represent the time span after entering the aviary. The vertical dashed line in panel **a** represents the start time for each experiment (i.e., Minutes = 0). In panel **b**, the regression line represents the conditional effect of air temperature on body temperature using the model-averaged regression estimates from the 95% confidence set of the best-ranked models (see Table 4).

**Figure 4:**
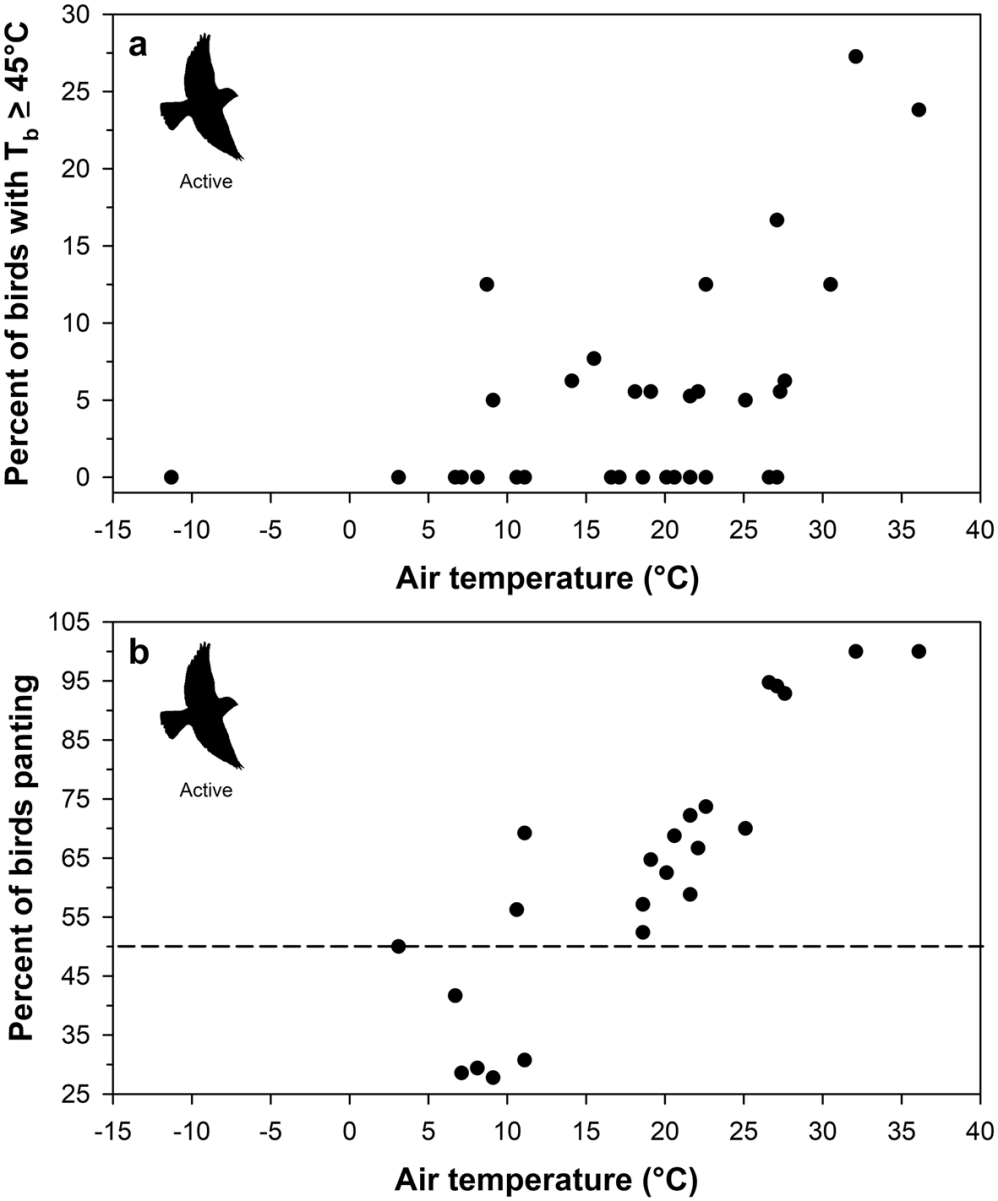
The impacts of air temperature on **a** the percent of actively flying snow buntings (*Plectrophenax nivalis*) with a body temperature (T_b_) greater than or equal to 45 °C and **b** the percent of buntings exhibiting panting behavior. Note the y-axes are on different scales. The horizontal dashed line in **b** represents the threshold above which 50% of the study population was dissipating heat through panting behavior. There are fewer data points in **b** as panting was only recorded during 2020.

**Figure 5:**
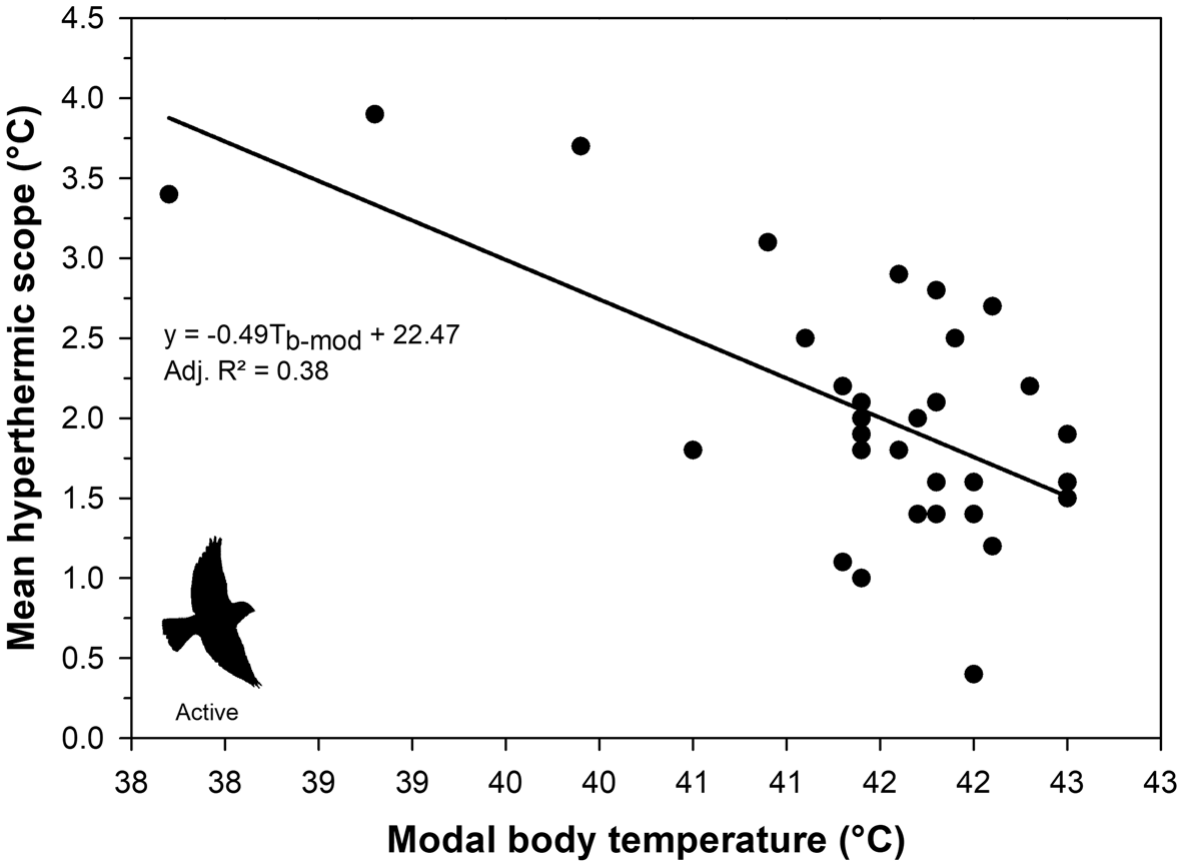
Mean hyperthermic scope (i.e., maximum flight body temperature—modal body temperature) as a function of modal body temperature (T_b-mod_) among actively flying snow buntings (*Plectrophenax nivalis*).

Active buntings showed a clear and immediate increase in body temperature during experimental flight trials relative to pre-experiment (calm) values (Fig. 3a, Table 2). The top candidate model explaining variation in active body temperature among buntings had strong support, being 10 × more likely relative to the next best model (Akaike weight of 80.33% vs. 8.3%; Table 3). Air temperature, body mass and their interaction were the most important predictors given their large effect sizes, large summed Akaike weights, and small *P-*values (Table 4). Body temperature increased linearly with air temperature. In 30 of the 36 flight experiments, 11 birds (4 intraperitoneal and 7 subcutaneous) had body temperatures ≥ 45 °C (Fig. 3b). Additionally, heavier birds exhibited a faster increase in T_b_ with T_a_. Even at air temperatures as low as 9°C, buntings were already exhibiting body temperatures of 45 °C or higher (Fig. 3b and Fig. 4a). At air temperatures from 3.1 to 11.1 °C, 27% to 69% of active birds were panting, and more than 50% of active birds were always panting once air temperatures exceeded 18.6 °C (Fig. 4b). Finally, the extent of hyperthermia in buntings was strongly dependent on an individual’s modal body temperature (Estimate = − 0.49 ± 0.11 °C, *P* = 0.0001; Fig. 5), with mean hyperthermic scope being greater in individuals with lower modal body temperatures.

**Table 2:** Mean ± standard error body temperature (T_b_), maximum T_b_ (T_b-max_) and the difference between T_b_ and modal body temperature (T_b_ − T_b-mod_) across snow buntings (*Plectrophenax nivalis*) with intraperitoneal or subcutaneous implants approximately 30 min before experimental flight trials (i.e., calm birds) and during flight experiments (i.e., actively flying birds).

Variable	Pre-experiment		Experiment	
	Intraperitoneal	Subcutaneous	Intraperitoneal	Subcutaneous
Mean T_b_	41.8 ± 0.6 °C (40.7–42.6 °C)^a^	41.4 ± 0.6 °C (39.8–42.5 °C)	43.5 ± 0.8 °C (42.3–44.6 °C)	43.4 ± 0.8 °C (41.1–44.8 °C)
Mean T_b-max_	42.4 ± 0.5 °C (41.7–43.2 °C)	42.3 ± 0.6 °C (41.4–44.0 °C)	44.6 ± 1.1 °C (43.2–46.9 °C)	44.9 ± 0.9 °C (43.1–47.0 °C)
Mean T_b_ − T_b-mod_	0.1 ± 0.7 °C (− 0.5–2.3°C)	0.1 ± 0.7 °C (− 0.7–2.1 °C)	1.9 ± 0.9 °C (0.4–3.7 °C)	2.1 ± 0.7 °C (1.1–3.9°C)

Values in parentheses represent the range of values for individual birds.

^a^Represents the range of mean body temperatures for individiual birds.

**Table 3.** 95% confidence set of the best-ranked regression models explaining variation in body temperature among snow buntings (*Plectrophenax nivalis*) during activity experiments.

Top Models	df	logLik	AICc^a^	ΔAICc	*w*_i_^b^	acc *w*_*i*_^c^	ER^d^
T_a_ + M_b_ + IP/SQ + T_a_:IP/SQ + T_a_:M_b_	8	− 698.3	1412.9	0.00	0.803	0.803	–
T_a_ + M_b_ + IP/SQ + AD + T_a_:IP/SQ + T_a_:M_b_	9	− 699.6	1417.4	4.52	0.083	0.886	9.8
T_a_ + M_b_ + T_a_:M_b_	6	− 703.5	1419.1	6.19	0.036	0.922	22.3

Fixed effects in the top candidate models included air temperature (T_a_), body mass (M_b_), intraperitoneal *vs.* subcutaneous implants (IP/SQ), the time T_b_ was measured in minutes relative to entering the aviary (i.e., activity duration; AD), and their interactions.

^a^Akaike’s information criterion adjusted for small sample sizes.

^b^Akaike’s weight. Indicates the probability that a given model is the best approximating model (Symonds and Moussalli^43^).

^c^The accumulated Akaike’s weights.

^d^Evidence ratio. Indicates how much better the best approximating model is relative to a given model based on Akaike’s weights (Symonds and Moussalli^43^).

**Table 4:** Standardized, model-averaged regression estimates from the 95% confidence set of the best-ranked models explaining variation in body temperature among snow buntings (*Plectrophenax nivalis*) during activity experiments.

Variable	Estimate (± SE)	95% CI	Summed weight^a^	*P*—value
Intercept	43.4 ± 0.1	43.1–43.7	*NA*	< 0.0001
T_a_	0.70 ± 0.06	0.58–0.82	1	< 0.0001
M_b_	− 0.90 ± 0.09	− 1.08 to − 0.73	1	< 0.0001
IP/SQ	0.39 ± 0.30	− 0.17–0.99	0.96	0.190
AD	0.006 ± 0.029	− 0.060–0.201	0.09	0.822
T_a_:M_b_	0.73 ± 0.14	0.47–1.00	1	< 0.0001
T_a_:IP/SQ	− 0.42 ± 0.15	− 0.70 to − 0.18	0.96	0.007

Predictors included air temperature (T_a_), body mass (M_b_), intraperitoneal *vs.* subcutaneous implants (IP/SQ), and the time T_b_ was measured in minutes relative to entering the aviary (i.e., activity duration; AD).

^a^The summed Akaike’s weights from each model in which that input variable is present. A value of 1 indicates that that variable was in every model within the 95% confidence set of the best-ranked models.

## Discussion

The interaction between activity and environmental temperature can significantly impact animal body temperature, thermoregulatory demands, subsequent behaviour and performance (see reviews by^12,46^). Recently, studies from hot, arid climates have demonstrated substantial costs to body condition and breeding success arising from behaviourial trade-offs between dissipating heat and performing essential activities^47–51^. Similar data for cold-climate species remains scarce, despite the rapid and unprecendent increases in air temperatures in the Arctic^14^. Here, we help fill this gap for a cold-specialist bird, by measuring body temperature increases of active snow buntings in relation to their calm body temperature across a range of environmental temperatures. Specifically, we wanted to determine how maximum body temperature correlated with duration of intense activity under warm air temperatures. At air temperatures of only 9 °C, snow buntings already had high body temperatures of 45 °C or above, and active buntings exhibited signs of heat stress (panting) at air temperatures of less than < 4 °C. These results confirm that even moderate air temperatures can significantly increase a snow bunting’s body temperature when they are active, which in turn increases heat dissipation requirements. Problematically, snow buntings posesses extremely poor evaporative cooling capacities. This was shown by O’Connor et al.^28^ who found remarkably low evaporative water loss rates during active panting in buntings experiencing rising temperatures. In fact, 95% of individuals were unable to dissipate an amount of heat equivalent to their own metabolic heat production. Snow buntings are thus expected to have to reduce their activity to avoid overheating when exposed to relatively mild temperatures^29^. As a result, our findings provide further evidence that as the Arctic continues to warm, snow buntings may increasingly need to rely on behavioural adjustments in activity to avoid high body temperatures during energetically expensive stages such as the nestling-provisioning period. Although the performance and fitness effects of behavioural responses to elevated temperatures we have reported in captive experiments still require empirical field-testing to confirm, the expectations are nonetheless potential costs from these behavioural adjustments to nestling condition and survival, potentially impacting breeding success.

The nestling-provisioning period for birds is considered one of the most energetically demanding life-history stages in altricial species^52^. As air temperatures increase under climate change, sustaining the optimal work levels required for feeding nestlings is predicted to become increasingly difficult as individuals are expected to experience higher body temperatures that would require increased heat dissipation behaviours^29^. Indeed, our data show that buntings regularly experienced higher body temperatures at higher air temperatures, frequently exceeding 45 °C, a value approaching lethal levels^7,8^. Similarly, Nilsson and Nord^53^ found that nestling-provisioning marsh tits (*Poecile palustris*) originating from a cool-temperate climate had higher body temperatures on days with higher ambient temperatures, with body temperatures also exceeding 45 °C on multiple occassions. The cause for increased body temperatures among buntings in response to increasing air temperature is likely two-fold. Firstly, snow buntings are cold-specialists having evolved traits for tolerating extreme cold^32^, possibly at the detriment for heat tolerance. Secondly, as air temperatures increase, the thermal gradient between body and air temperature becomes increasingly narrow, thereby restricting the flow of heat through non-evaporative pathways. Given that snow buntings are extremely limited in their capacity to evaporatively dissipate heat^28^, it becomes increasingly hard for them to dissipate heat at higher ambient temperatures. Moreover, buntings maintain cold hardiness even after arrival on their breeding grounds^31,54^. Collectively, these results underscore the likelihood that as the Arctic continues to warm at a rapid rate, nestling-provisioning adults performing at high sustained metabolic rates will increasingly experience periods where they may be unable to physiologically limit their body temperature from approaching lethal levels, requiring them to behaviourally allocate more time towards dissipating heat. As has recently been demonstrated for hot, arid bird species^10,12, 47^, behavioural adjustments are likely to result in downstream costs to essential parental activities (e.g., nestling provisioning) and possibly fitness^29^.

Predicting the temperature theresholds above which species will begin to experience fitness costs remains a significant challenge in predicting climate change impacts^55–57^. Although valuable, mechanistic models used to calculate mass and heat transfer rates among species are complex, requiring numerous physiological and morphological parameters. Recently, Smit et al.^10^ proposed a less computationally intense heat dissipation behaviour index for assessing the vulnerability of species to high environmental temperatures. Specifically, this index uses presence/absence data to calculate when heat dissipation behaviours (e.g., panting or wing spreading) occur in at least 50% of instances. Using this index, Smit et al.^10^ found that among 33 Kalahari desert bird species, the median air temperature at which panting occurred in 50% of observations ranged from approximately 30–46 °C. In stark contrast, we found that by air temperatures of 12–18 °C, more than half of our snow buntings were panting. The lower end of this air temperature range also aligns remarkably well with O’Connor et al.’s^29^ prediciton that buntings will have to start actively dissipating heat at an air temperature of only 11.7 °C, even though this prediction was for birds opperating at a lower metabolic rate than birds in our current study. Together, these findings suggest that buntings will need to increase their thermoregulatory (e.g., cooling) behaviours in response to increases in T_b_ at moderately low air temperatures, suggesting they will increasingly experience thermoregulatory demands (i.e., risk of overheating) on their Arctic breeding grounds into the future.

The regulation of body temperature can significantly influence the transfer of heat and thus affect an animals thermoregulatory demands^25,26, 58, 59^. Indeed, most birds have been shown to facultatively increase their body temperature when subjected to high air temperatures, an adaptive trait often attributed to enhancing water economy^4–6^. Consequently, although seemingly counterintuitive for heat tolerance, the regulation of a lower T_b_ may be advantageous in the presence of high air temperatures as an individual’s baseline T_b_ is further away from lethal levels, therefore lessening the risk of overheating when becoming hyperthermic^60^. Overwhelmingly, investigators have studied adaptive thermoregulation among populations in response to climatic variables (e.g., arid versus mesic), both at the inter-and intra-specific level^61–63^. However, to our knowledge no studies have reported intra-specific body temperature variation within the same population. Here, we found that mean body temperature and modal body temperature among snow buntings varied by 2.2 °C and 2.7 °C, respectively. Moreover, we found strong evidence that individuals with higher modal T_b_ at rest increased T_b_ less when active, suggesting that birds with a higher resting T_b_ (i.e., when calm) may have a reduced scope for further T_b_ increases when performing active portions of their life history. While further work is required to determine whether these differences in scope are fixed at the individual level, variation in a set-point body temperature within the same population could allow certain individuals to prolong activity at high air temperatures given the larger spare capacity for hyperthermia. Consequently, buntings with a lower set-point body temperature could have a selective advantage under a rapidly warming Arctic as they would be less at risk of overheating when working at peak levels^4,5^.

## Data Availability

The datasets generated during and/or analysed during the current study are available from the corresponding author on reasonable request.
